# Deworming of stray dogs and wild canines with praziquantel-laced baits delivered by an unmanned aerial vehicle in areas highly endemic for echinococcosis in China

**DOI:** 10.1186/s40249-017-0329-8

**Published:** 2017-06-27

**Authors:** Qing Yu, Ning Xiao, Shi-jie Yang, Shuai Han

**Affiliations:** 1 Department of Echinococcosis, National Institute of Parasitic Diseases, Chinese Center for Disease Control and Prevention, 207 Rui Jin Er Road, Shanghai, 200025 China; 2 Key Laboratory of Parasite and Vector Biology, National health and Family Planning Commission, Shanghai, China; 3 WHO Collaborating Center for Tropical Diseases, Shanghai, China

**Keywords:** Echinococcosis, Wild canine definitive hosts, Control: Unmanned aerial vehicle, Delivery, Baits, Qinghai-Tibet Plateau, China

## Abstract

**Background:**

Canines, the definitive hosts for the parasites causing alveolar (AE) and cystic echinococcosis (CE), are the main source of this infections playing the key role in the transmission. The ten-year mortality rate of AE is extremely high (94%) if the patients are not given sustained treatment. The aim of this field study is to explore the possibility of delivery of praziquantel-laced baits using unmanned aerial vehicles (UAVs) aimed at deworming wild canines in the endemic areas.

**Methods:**

UAVs were compared to manual bait delivery in the 1-km^2^ test areas followed by testing of canine faeces using an *Echinococcus* coproantigen ELISA test in the ensuing year. The outcomes of the two approaches were compared with respect to time of delivery and overall cost.

**Findings:**

Compared to manual bait delivery, delivery by UAVs saved up to 67% of the overall cost. Three times more staff was needed for the former approach compared to the latter and, time wise, UAV bait delivery saved 350% compared to manual bait delivery on average. With regard to investment needed, the use of UAVs showed an efficiency 2.5 times better than manual bait delivery. Compared to the area served by UAVs, the average positive rate for the canine faecal samples was more than 38% higher in the area served manually.

**Conclusion:**

The technique of bait delivery with praziquantel using UAVs for canine deworming has a strong potential with regard to savings of manpower, time and overall cost in areas highly endemic for echinococcosis.

**Electronic supplementary material:**

The online version of this article (doi:10.1186/s40249-017-0329-8) contains supplementary material, which is available to authorized users.

## Multilingual abstracts

Please see Additional file 1 for translations of the abstract into the six official working languages of the United Nations.

## Background

Cystic echinococcosis (CE), due to *Echinococcus granulosus*, and alveolar echinococcosis (AE), due to *E. multilocularis,* are caused by the larval cystic stage of these small tapeworms. Canines are the most common definitive hosts with herbivorous animals, such as sheep, cattle and goats as intermediate hosts. Humans are infected accidentally and do not transmit the infection further. Still, more than one million persons suffer from echinococcosis globally [1]. Due to the high disease burden and mortality (up to 94% within ten years if sustained treatment is not provided) of AE, this form of the disease has been called “the worm tumour” [1–4].

Echinococcosis (also called hydatid disease) is widely distributed in the pastoral and agriculture-pastoral parts of China, including the provinces and autonomous regions of Inner Mongolia, Sichuan, Yunnan, Tibet, Shaanxi, Gansu, Qinghai, Ningxia, and Xinjiang where canines constitute the main source of infection. A national epidemiological survey in China carried out in 2012 showed an average prevalence rate of this infection in dogs of 4.3% by a coproantigen ELISA test, whereas it reached 70% prevalence among stray/wild dogs in parts of Yushu Tibetan Autonomous Prefecture of Qinghai Province [5–8]. Baits laced with praziquantel (PZQ) for control of the adult form of *E. granulosus* in wild canids have been used effectively in areas endemic for AE in Europe [9–11]. Measures based on monthly dog treatment, executed by the control programme in western China since 2006, produced good results, both for CE and AE control [12, 13]. This approach showed that anthelmintic monthly PZQ treatment for dogs could eliminate disease transmission simply due to the fact that the interval between treatments is then shorter than the time required for the maturation and start of egg laying (45 days) of the parasites *E. multilocularis* and *E. granulosus* [14, 15]. However, human CE and AE are still highly endemic in China with AE patients accounting for 22.4% of the total number of patients according to the latest national survey [4].

The parasites are multiple-host pathogens with passage between humans and livestock as a part of its natural circulation. This situation is exacerbated by the fact that humans commonly keep many different domestic animals, which are part of the parasite life cycle together with tame and wild canines. Wild canines in particular play a crucial role as definitive hosts in AE transmission characterised by wide distribution, large numbers and complex classification. To date, unavailable and/or inefficient interventions make it difficult to block the transmission in strongly endemic areas [16–20].

In China, echinococcosis is mainly co-endemic in the western region, particularly on the Qinghai-Tibet Plateau, where incidence and the number of people at risk rank one of the highest in the world (9). Furthermore, this disease threatens local farmers and herdsmen and is widely spread throughout the Shiqu County of Ganzi Tibetan Autonomous Prefecture in Sichuan Province, where both CE and AE are a severe public health concern with control considered extremely difficult, of AE in particular, due to the wild canine population [4, 21, 22].

Satellite-based remote-sensing and aerial photography would be useful in finding biotopes suitable for *Echinococcus* transmission. The introduction of drone photography could be significantly useful here as it does not only reach inaccessible areas, but also reduces time and cost of data acquisition. Unmanned aerial vehicles (UAVs) are lightweight, have a diminutive size and can easily be efficiently manoeuvred over specified ranges, both small and large. They have been extensively applied for reconnaissance and inspections (railways, bridges, and roads) and also used to support agriculture and investigate environmental pollution and health in general [23–26]. Apart from discussions on the advantages and disadvantages of UAVS applications *vis-à-vis* remote-sensing for the collection of spatial, epidemiological data, there are not many studies focused on specific infectious diseases [27, 28].

Here, we explore the use of UAVs for the distribution of baits laced with PZQ for blocking CE and AE transmission by deworming wild canine populations in a highly endemic area of echinococcosis in the Qinghai-Tibet Plateau.

## Methods

The present work took place under the auspices of the National Institute of Parasitic Diseases (NIPD), Chinese Center for Disease Control and Prevention (China CDC) as a trial in an area highly endemic for echinococcosis. A study area endemic for echinococcosis was chosen in GeMeng Town, Shiqu County in the Ganzi Tibetan Autonomous Prefecture in Sichuan.

### Study area

Two pilot areas located at an average altitude of 4300 m in GeMeng Town, Shiqu County where wild canines (stray dogs, foxes, and wolves) roam free in close contact with livestock was recommended by local residents. One area was used for manual bait delivery and the other for UAV delivery. A mobile global positioning system (GPS) device (Model: GPSMAP 629SC) was used to locate and synchronize the point for bait distribution.

**Fig. 1:**
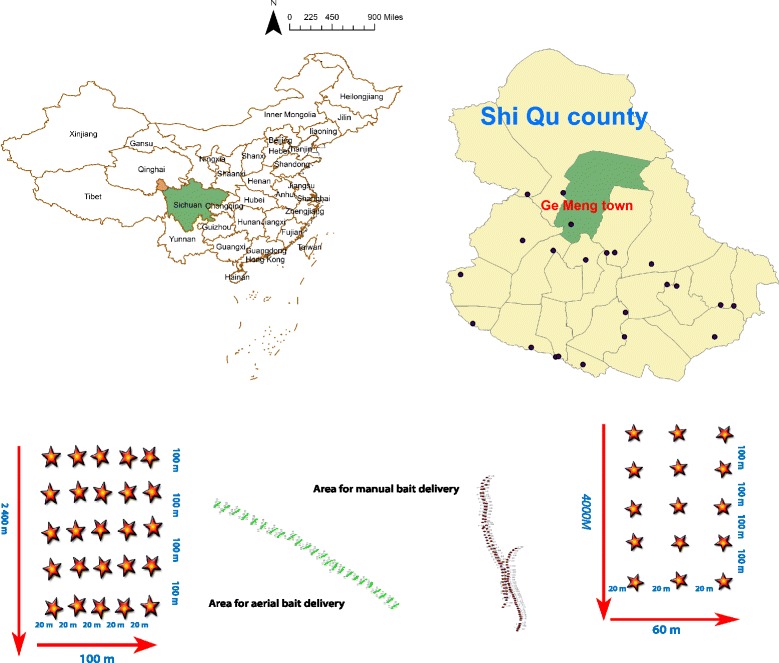
Area distribution for aerial and manual bait delivery. The bait was distributed in every 20 × 100 m cross point over the total coverage area of 0.48 km^2^ with 0.24 km^2^ for each area

**Fig. 2:**
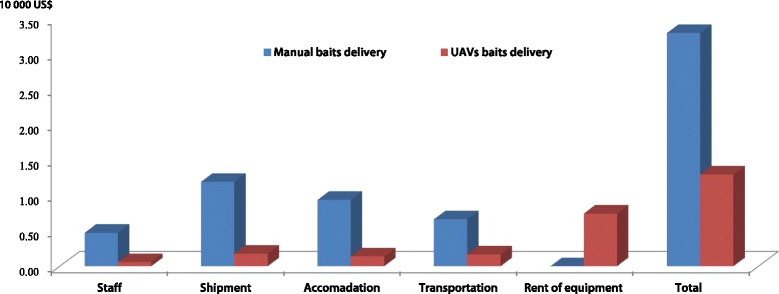
Cost for baits delivery between two groups with coverage area of 1 km^2^ (by estimation)

The total coverage was 0.48 km^2^ divided into 0.24 km^2^ for each area, which in turn were divided into units of 20 (latitudinal distance) × 100 (longitudinal distance) meters containing fixed points for bait delivery. The baits were distributed in every cross point by 20 × 100 m meaning that a total of 240 fixed points were set up and recorded by the GPS instrument (Figs. 1 and 2).

### Study design

Beads with a diameter of 13 mm containing an effective dose of 50 mg PZQ (Nanjing Pharmaceutical Co., Ltd., Jiangsu Province, China) were used as bait. As tame dogs are normally dewormed with an effective dose of 200–400 mg PZQ depending on weight, we used eight beads as bait (400 mg PZQ in total) in each place.

The baits were distributed manually in one of the two study areas and by an UAV in the other. The UAV (model 4DE1000) was rented from an independent company (Jiangsu AI Jin agrochemical Co., Ltd., Jiangsu Province, China) and modified by them for the bait dosage delivery. The UAV had multi-rotors giving the device a flight radius of 200 m with a load up to 5 kg. Other characteristics included a 3–5 m/s climb rate, 20–36 km/h flight speed, 4–5 grade speed wind resistance and 20 min flight time.

Faecal samples were collected at the same places where the baits were delivered in both study areas every 2 months in 2016 from April to October. The type of animal that had produced the faecal samples was identified with the aid of local residents and tested for *Echinococcus* antigen using a commercially available coproantigen ELISA test (Shenzhen Combined Biotech Co., Ltd., Guangdong Province, China.).

### Statistics and records

Maps covering the study area were produced using ArcGIS software version 10 (ESRI, Redlands, CA, USA). Fisher’s exact test was used for the statistical analysis that was performed using Microsoft Excel software version 2010 and SPSS Statistics v18.0 (Statistical Package for the Social Sciences. SPSS Institute, Chicago, IL, USA). A statistically significant difference was defined as a *P*-value <0.05.

During the whole process, labour and time for bait delivery and costs for staff, transportation, UAV rent and fuel, were recorded, statistically analyzed and calculated using the exchange rate of 6.5 RMB Yuan per USD.

## Results

### Labour and time consumption

In the study area where the UAV was used, two staff spending 60 min (recorded by stopwatch) was needed for the task, while six staff working for 270 min had to be hired for the manual operation in the other area. Thus, only a third of the manpower rate for manual delivery was needed for UAV delivery. In addition, the latter approach saved 350% [(270–60)/60] of the time compared to manual bait delivery.

### Cost of baits delivery

Comparing the cost between the two groups for bait delivery over the one km^2^ area (equal to one million m^2^), the results showed that the expenditure was 3.29 USD/100 m^2^ in the manual area against 1.30 USD/100 m^2^ using UAV delivery. Thus, the use of UAVs saved approximately 60.5%[(3.29–1.30)/3.29] of the financial expenditure, i.e. the final cost was more than 2.5 times better with UAV delivery (Table 1).

**Table 1:** Cost of baits delivered manually *vis-à-vis* unmanned aircraft vehicle delivery

Type of bait delivery	Area (m^2^)	Staff	Ship-ment	Accommo-dation	Transpor-tation	Rent of equipment	Total (USD × 10^4^)
UAV^a^	10^6^	0.06	0.18	0.14	0.17	0.75	1.30
Manual	10^6^	0.48	1.20	0.94	0.67	0.00	3.29

^a^Unmanned aircraft vehicle

### Deworming outcome

A total of 464 faecal samples were collected from the 240 fixed points in the two study areas. No significant difference between two areas was found (*χ* ^*2*^ = 0.09, *P* > 0.05). However, it was noted that the life cycle of *E. granulosus* included mainly a dog-sheep-dog cycle but goats, swine, horses, cattle, camels, yaks and other domestic animals were also involved, while that of *E. multilocularis* also involved foxes, other carnivores and small mammals (mostly rodents). In both our study areas, the number of faecal samples of foxes found ranked at the top followed by stray dogs with wolves in the third place. Moreover, the average infection rate based on the coproantigen ELISA was 38.2%[(1.52–1.10)/1.10] higher in the manual area than in the one served by the UAVs, likely attributed to the different probability for baits uptake by wild canids in manual and UAV areas, as well as, to the uneven density of small mammals whose populations fluctuate tremendously (Table 2).

**Table 2:** Test results for *Echinococcus* antigen in faecal samples after praziquantel-laced baits delivered manually *vis-à-vis* unmanned aircraft vehicle delivery

Type of bait delivery	Time of faecal collection	Number of samples	Proportion of faeces from wild animals (%)^a^			Positive rate^b^
			Stray dogs	Foxes	Wolves	%
Unmanned aircraft vehicle (UAV)	April	60	20.83	25.00	54.17	1.67
	June	55	55.17	37.93	6.90	1.82
	August	66	14.71	64.71	20.59	0.00
	October	36	0.00	89.47	10.53	0.00
Total		217	24.53	52.83	22.64	1.10
Manual	April	75	56.90	36.21	6.90	1.33
	June	73	36.00	56.00	8.00	1.37
	August	50	2.86	77.14	20.00	2.00
	October	49	41.18	41.18	17.65	0.00
Total		247	37.04	51.11	11.85	1.52
Grand total		464	31.19	51.92	16.90	1.08

^a^Identified by shape; ^b^Coproantigen ELISA test

## Discussion

As far as we are aware this is the first study of the use of UAVs for the distribution of praziquantel-laced baits for the control of echinococcosis. Our results show that considerable costs, as well as time, can be saved by this approach.

The type of animals found to play the role of definite hosts in CE and AE transmission were those mentioned in other reports [9, 28]. Though prevention and control of AE is particularly complex since the parasite’s life cycle involves wild animal species as both definitive and intermediate hosts, distribution of anthelmintic baits against wild and stray definitive hosts results in significant reductions in AE prevalence. For example, the risk for AE in Germany has been pointed out [10, 11] with a field study in southern Germany indicating reduced *E. multilocularis* prevalence in red foxes after anthelmintic bait delivery [29]. In northern Japan, a bait-delivered anthelmintic also reduced the prevalence of this parasite in red foxes putting forward a discussion of optimizing anthelmintic ways in a more cost-effective manner [30–32]. As it is already clear that treatment of animals is a successful approach for echinococcosis control, with special reference to wild carnivores in the case of AE, the main focus of our study was to evaluate a new cost-effective technique for the anthelmintic bait delivery.

The finding that foxes and stray dogs rank at the top in terms of faecal matter found in the field indicate that canids have wide distribution in rural environments though we need more proof to confirm this by further research on wild canine activities, including observation and genetic detection of the faecal samples. Another limitation of this study was the short duration of observation that made it difficult to show the long-term positive effect of canid deworming beyond doubt. Still, it is already obvious that deworming wild and tame canids through PZQ-laced baits delivered by UAVs saves both cost and labour.

## Conclusion

The technique of baits with praziquantel delivery using UAVs for canine deworming has the potential to save cost and labour in areas highly endemic for echinococcosis. This has been shown to be true in Qinghai-Tibet Plateau and should work equally well also in other areas.

## Additional files

Additional file 1: Multilingual abstracts in the six official working languages of the United Nations. (PDF 633 kb)

Additional file 2: Baits delivery by UAVs. (MP4 74,029 kb)

## Abbreviation

UAVs: Umanned aircraft vehicles
