# Supplementary material for: Deworming of stray dogs and wild canines with praziquantel-laced baits delivered by an unmanned aerial vehicle in areas highly endemic for echinococcosis in China
Source: Infect Dis Poverty. 2017 Jun 27;6:117. doi: 10.1186/s40249-017-0329-8 (PMC5488432; doi:10.1186/s40249-017-0329-8)
Supplement: Supplementary file 1 — Multilingual abstracts in the six official working languages of the United Nations. (PDF 633 kb) [file 40249_2017_329_MOESM1_ESM.pdf]

Translation of the abstract into the six official working languages of the United Nations

إزالة الديدان من الكلاب الضالة والكلاب البرية مع الدواء (الطعوم) برازيكوانتيل لاسد هذه الطعوم يتم تسليمها من قبل مركبة جوية بدون طيار في المناطق المتوطنة للغاية لمرض إكينوكوكاس (echinococcosis) في الصين

Qing Yu, Ning Xiao, Shi-jie Yang, Shuai Han

المستخلص

المقدمة: الأناضول، العوائل النهائية للطفيليات التي تسبب السنخية (alveolar) والمكورات المشيمة الكيسي (cystic echinococcosis)، هي المصدر الرئيسي لهذه العدوى والتى تلعب الدور الرئيسي في انتقال العدوى. معدل وفيات عشر سنوات من السنخية عالية للغاية (94٪) إذا لم يتم إعطاء المرضى العلاج المستمر. والهدف من هذه الدراسة الميدانية هو استكشاف إمكانية تسليم الطعوم برازيكوانتيل لاسد باستخدام الطائرات بدون طيار, تهدف إلى إزالة ديدان الكلاب البرية في المناطق المتوطنة.

الطريقة: تمت مقارنة تسليم الطائرات بدون طيار للطعم مع تسليم الطعم يدوياً في مناطق الاختبار 1 كم^2 تليها اختبار براز الكلاب باستخدام إيشينوكوكوس كوبروانتيجن (*Echinococcus* coproantigen) باختبار الايلزا (ELISA) في السنة التي تلت ذلك. وتمت مقارنة نتائج النهجين فيما يتعلق بوقت التسليم والتكلفة الإجمالية.

النتائج: بالمقارنة مع تسليم الطعم يدوياً، تسليم الطائرات بدون طيار حفظ ما يصل إلى 67٪ من التكلفة الإجمالية. كان هناك حاجة إلى ثلاثة أضعاف من الموظفين للنهج السابق مقارنة مع الأخير، وكذلك عامل الوقت، في المتوسط توفير الطعم بالطائرات بدون طيار حفظ 350٪ مقارنة مع تسليم الطعم اليدوي. وفيما يتعلق بالاستثمار المطلوب، أظهر استخدام الطائرات بدون طيار كفاءة 2.5 مرة أفضل من تسليم الطعم اليدوي. وبالمقارنة مع المنطقة التي تخدمها الطائرات بدون طيار، كان متوسط المعدل الإيجابي لعينات براز الكلاب أكثر من 38٪ أعلى من المنطقة التى تخدم يدوياً.

الخلاصة: تقنية تسليم الطعم مع برازيكوانتيل باستخدام الطائرات بدون طيار لديدان الكلاب كان له امكانات قوية فيما يتعلق بتوفير القوى العاملة والوقت والتكلفة الإجمالية في المناطق المتوطنة للغاية للمكورات المشيمة.

Translated from English version into Arabic by Khaled Zayed

中国棘球蚴病重度流行区应用无人机投放吡喹酮驱虫药饵作用于流浪犬及野外犬科类动物可行性观察

余晴，肖宁，杨诗杰，韩帅

摘要

引言：犬科类动物是造成人类囊型和泡型包虫病流行且公认的最主要传染源，其中泡型包虫病危害最大，未经治疗的泡型包虫病患者 10 年死亡率高达 94%。本次现场研究目的是在包虫病重度流行区探索应用无人机投放吡喹酮驱虫药饵作用于野外犬科类动物的可行性，为传染源控制提供技术手段。

方法：在 1 平方公里范围内，比较人工和无人机两种投药方式的作业时间和全部投入成本，同时采用 ELISA 粪抗原检测方法对研究区域内的捡获的犬科类动物野粪进行检测，了解粪抗原阳性率变化。

结果：研究发现，同人工投药方式相比，采用无人机技术可以节约 67%经济总成本，可节省 3 倍或更多的人力投入，平均可节约 350%以上的作业时间且工作效率是人工的 2.5 倍。此外，野粪检测结果显示，人工投药区域野粪抗原阳性率高出无人机投药区域 38%。

结论：在包虫病流行区犬科类传染源控制进程中，应用无人机投放吡喹酮驱虫药饵作用于野外犬科类动物技术无论在人力节省，时间节约及成本控制上具有很好地潜在应用前景。

Translated from English version into Chinese by Qing Yu

Déparasitage de chiens errants et de canidés sauvages en utilisant des appâts à praziquantel émis par un drone dans les régions de Chine où l'échinococcose est fortement endémique

Qing Yu, Ning Xiao, Shi-jie Yang, Shuai Han

Résumé

Contexte: Les canidés, hôtes définitifs des parasites responsables de l'échinococcose alvéolaire (EA) et kystique (EK), sont la principale source de cette infection jouant ainsi un rôle essentiel dans la transmission. Le taux de mortalité décennale de l'EA est extrêmement élevé (94%) si les patients ne sont pas soumis au traitement prolongé. Le but de cette étude est d'explorer la possibilité d'utiliser des appâts à praziquantel distribués par un drone communément appelé véhicule aérien télépiloté visant au déparasitage des canidés errants dans les régions endémiques.

Méthodes: Les drones ont été comparés à la distribution manuelle d'appât dans des zones d'essai de 1-km^2 de surface suivi de l'analyse des fèces de canidés en utilisant un test ELISA de détection de cropoantigen à *Echinococcus* dans l'année suivante. Les résultats des deux approches sont comparés en considérant le temps de distribution et le coût global investi.

Découvertes: Comparé à la distribution manuelle d'appât, le drone a fait économiser jusqu'à 67% de l'ensemble des coûts. Un nombre estimé à trois fois plus de personnel est nécessaire pour la première approche et, en timing, la distribution d'appât par drone a fait économiser en moyenne 350% par rapport à celle faite manuellement. Quant à ce qui concerne les investissements requis, l'utilisation de drones a montré une efficacité 2,5 fois meilleure que la dristribution manuelle. En comparaison à la région desservie par les drones, le taux moyen de tests positifs des échantillons fécaux de canidés était plus de 38% élevé que celui dans la zone desservie manuellement.

Conclusion: La technique de dristribution d'appât à praziquantel avec l'utilisation de drones comme moyen de déparasitage des canidés a un fort potentiel vu la réduction de la main d'œuvre, du temps et du coût global d'investissement dans les régions où l'échinococcose est fortement endémique.

Translated from English version into French by Kokouvi Kassegne

Дегельминтизация собак бродячих и диких собак с празиквантелом-ажурными прибалтами доставлены беспилотным летательным аппаратом в районах, высокоэндемичных для эхинококкоза в Китае

Qing Yu, Ning Xiao, Shi-jie Yang, Shuai Han

Аннотация

Введение: Клыки, это окончательный хозяин для паразитов и вызывает альвеолярный (AE) и кистозный эхинококкоз (CE), которые являются основным источником этой инфекции и играет ключевую роль в передаче. Десятилетняя смертность AE чрезвычайно высока (94%), если

пациенты не получают постоянное лечение. Цель данного исследования поля состоит в том, чтобы изучить возможность поставки празиквантела-ажурных приманк с использованием беспилотных летательных аппаратов (UAVs) с целью дегельминтизации диких собак в эндемичных районах.
Методы: UAVs по сравнению с ручной доставки приманки в 1-km^2 зонах испытаний с последующим тестированием собачьих фекалий с помощью теста ELISA *Echinococcus* coproantigen в следующем году. Результаты двух подходов по сравнению с учетом времени доставки и общей стоимости.
Выводы исследования: По сравнению с ручной доставкой приманки, которая была поставленна UAVs и сохраняется до 67% от общей стоимости. Выросло число персонала в три раза для бывшего подхода по сравнению с последним и, так, доставка приманки UAV сэкономила 350% по сравнению с ручной доставки приманки в среднем. Что касается необходимых инвестиций, использование UAVs показало эффективность в 2,5 раза лучше, чем эффективность ручной доставки прикормки. По сравнению с областью, которая обслуживается UAVs, средний положительный показатель для образцов собачьих фекалий был более чем в 38% выше в области, которая обслуживается самостоятельно.
Заключение: Метод доставки прикормки с празиквантелем с использованием UAVs для дегельминтизации собак имеет большой потенциал с точки зрения экономии сил, времени и общего затрата в районах высокоэндемичных по эхинококкоза.

Translated from English version into Russian by Hao-Qi Zhang

Desparasitación de perros callejeros y caninos salvajes con praziquantel adherido a carnadas esparcidos por un vehículo aéreo no tripulado en áreas altamente endémicas para la equinococosis en China

Qing Yu, Ning Xiao, Shi-jie Yang, Shuai Han

Abstracto

Trasfondo: Los caninos, los hospederos definitivos de los parásitos causantes de la equinococosis alveolar (EA, por sus siglas en inglés) y quística (CE, por sus siglas en inglés), son la principal fuente de estas infecciones desempeñando el papel clave en la transmisión. La tasa de mortalidad a diez años de AE es extremadamente alta (94%) si los pacientes no reciben tratamiento sostenido. El objetivo de este estudio de campo es explorar la posibilidad de la entrega de carnadas adheridas a praziquantel utilizando vehículos aéreos no tripulados (UAVs, por sus siglas en inglés) dirigidos a la desparasitación de caninos salvajes en las áreas endémicas.
Métodos: Los UAVs se compararon con el suministro manual de las carnadas en las áreas de prueba de 1 km^2, seguido de la prueba de heces caninas utilizando una prueba ELISA de coproantígenos de Echinococcus en el año siguiente. Los resultados de los dos enfoques se compararon con respecto al tiempo de entrega y el costo total.
Hallazgos: En comparación con la entrega de la carnada manual, la entrega por UAVs ahorró hasta un 67% del costo total. Tres veces más personal era necesario para el enfoque anterior en comparación con

el último y, en el tiempo, la entrega de carnada mediante UAVs ahorró un 350% en comparación con la entrega de la carnada manual en promedio. Con respecto a la inversión necesaria, el uso de vehículos aéreos no tripulados mostró una eficiencia 2.5 veces mejor que la entrega manual de la carnada. En comparación con el área atendida por UAVs, la tasa positiva promedio para las muestras fecales caninas fue más del 38% mayor que en el área servida manualmente.

Conclusión: La técnica de entrega de cebo con praziquantel utilizando UAVs para la desparasitación canina tiene un fuerte potencial con respecto al ahorro de mano de obra, tiempo y costo total en áreas altamente endémicas para la equinococosis.

Translated from English version into Spanish by Laura C Vicente Rodriguez
